# Genomic Diversity of Human Respiratory Syncytial Virus (HRSV) Type A in a Pediatric Cohort in the United States After the Onset of the COVID-19 Pandemic

**DOI:** 10.3390/microorganisms14081656

**Published:** 2026-07-29

**Authors:** Luciane Amorim Santos, Anastasia Castillo, Emanuele Gustani-Buss, Matt D. T. Hitchings, Peiyu Liu, Nicole Iovine, Melanie N. Cash, Jose L. Estrada, Laise de Moraes, Philippe Lemey, John Lednicky, John Glenn Morris, Kathleen Ryan, Marco Salemi

**Affiliations:** 1 Instituto Gonçalo Moniz, Fundação Oswaldo Cruz, Salvador 40296-710, Bahia, Brazil; lucianeamorim@bahiana.edu.br (L.A.S.); laise.moraes@fiocruz.br (L.d.M.); 2 Escola Bahiana de Medicina e Saúde Pública, Salvador 40290-000, Bahia, Brazil; 3 Department of Pediatrics, College of Medicine, University of Florida, Gainesville, FL 32611, USA; anastasia.castillo@nemours.org; 4 Department of Microbiology and Immunology, KU Leuven, 3000 Leuven, Belgium; e.c.gustanibuss@umcutrecht.nl (E.G.-B.); philippe.lemey@kuleuven.be (P.L.); 5 Department of Pediatric Immunology and Infectious Diseases, University Medical Centre Utrecht, 3584 CX Utrecht, The Netherlands; 6 Emerging Pathogens Institute, University of Florida, Gainesville, FL 32611, USA; mhitchings@ufl.edu (M.D.T.H.); peiyu.liu@ufl.edu (P.L.); nicole.iovine@medicine.ufl.edu (N.I.); mcash@pathology.ufl.edu (M.N.C.); joser3@ufl.edu (J.L.E.); jlednicky@phhp.ufl.edu (J.L.); jgmorris@epi.ufl.edu (J.G.M.J.); 7 Department of Biostatistics, College of Public Health and Health Professions, University of Florida, Gainesville, FL 32611, USA; 8 Office of Infection Control, UFHealth, Gainesville, FL 32611, USA; 9 Department of Environmental and Global Health, College of Public Health and Health Professions, University of Florida, Gainesville, FL 32611, USA; 10 Department of Pathology, Immunology and Laboratory Medicine, University of Florida, Gainesville, FL 32611, USA

**Keywords:** respiratory syncytial virus, pediatrics, public health surveillance, phylogeography, virus evolution

## Abstract

Human Respiratory Syncytial Virus (HRSV) is a leading cause of respiratory infections in young children. HRSV cases declined dramatically in the initial stages of the COVID-19 pandemic, followed by a resurgence in 2022/2023. To investigate patterns of viral genetic diversity and evolutionary dynamics during this period, HRSV sequences were analyzed at national and global levels, including newly sequenced samples from pediatric patients at a major Florida medical center in Alachua County. Our Florida strains clustered within multiple clades, with no clear association between specific clades and clinical or epidemiologic characteristics. Phylodynamic analyses suggested post-pandemic changes in HRSV-A and HRSV-B population dynamics, while phylogeographic patterns were compatible with possible viral introductions from European countries. We also detected amino acid diversity in known G-protein epitope regions, indicating a genetic variation in antigenically relevant sites. These findings suggest that post-pandemic HRSV circulation was associated with changes in viral population dynamics and genetic diversity, emphasizing the value of continued genomic surveillance to monitor viral evolution and support future prevention strategies.

## 1. Introduction

Orthopneumovirus hominis (Human Respiratory Syncytial Virus—HRSV) is a leading cause of acute lower respiratory tract infections worldwide, affecting infants and young children as well as older adults. HRSV is spread through direct contact and by airborne/aerosol routes of transmission, with primary infection of respiratory mucosa and, in children, is often manifested as bronchiolitis with respiratory distress, hypoxemia, cough, congestion, and apnea [1]. Despite advances in our understanding of HRSV epidemiology and pathogenesis, the virus continues to pose a substantial public health burden: the United States Centers for Disease Control and Prevention estimate that among children younger than 5 years of age in the United States (US) HRSV is responsible for some 2.1 million outpatient visits and 58,000–80,000 hospitalizations per year [2]. In the US, the HRSV season typically begins in the fall and peaks in the winter, often following a biennial oscillation pattern [3]. In Florida, HRSV cases tend to occur earlier and last longer than in other parts of the United States, with cases/outbreaks occurring year-around in southeast Florida and an HRSV season that extends from August to March in Central Florida [4].

The COVID-19 pandemic resulted in substantial disruption of the typical seasonal pattern of HRSV cases, with an unprecedented decline in cases during the initial epidemic period followed in 2022/2023 by a striking resurgence in case numbers, with increases in hospitalization rates at some sites in excess of 80% [5,6,7,8,9]. It has been hypothesized that the initial decrease in HRSV transmission was associated with implementation of non-pharmacologic interventions (handwashing, masking, social distancing, lockdowns) at the beginning of the COVID-19 pandemic, in keeping with patterns seen with other respiratory viruses such as influenza, human metapneumovirus, and parainfluenza [10]. Reasons for the subsequent upsurge in cases, after most COVID-19 interventions had been dropped, are less well understood. It has been proposed that this was a function of “immunity debt,” with the lack of immune stimulation by viruses for prolonged periods of time having increased the pool of immunologically vulnerable children [3]. Although there were concerns about the potential emergence of new, more virulent clones, this has not been borne out by genomic studies which have linked cases in the 2022/2023 time window with lineages observed globally for more than a decade [3,6,11]. Nevertheless, fundamental questions remain about how the pandemic may have affected global genomic patterns and how these changes impact future HRSV transmission. Understanding this is essential for implementing effective control and prevention strategies.

Herein, we characterize the genetic diversity and dispersal dynamics of HRSV-A and HRSV-B strains at the US and global level, including newly sequenced strains from Alachua County, Florida pediatric cases between August 2022 and March 2023. Specifically, we aimed to determine whether post-pandemic HRSV circulation was associated with shifts in population dynamics and increased amino acid variability within known protein epitope regions. Our phylodynamic analysis reveals that, following the COVID-19 pandemic, both HRSV types experienced changes in the frequency of population bottleneck events. These changes coincided with increased amino acid variability within known G-protein epitope regions, emphasizing post-pandemic shifts in HRSV population dynamics and antigenically relevant genomic regions that warrant continued surveillance.

## 2. Materials and Methods

### 2.1. Study Design and Population

This retrospective population-based cohort study included 48 pediatric patients aged 0–18 years with symptomatic HRSV bronchiolitis who were seen at UFHealth Children’s Hospital within the University of Florida Health System at Alachua County, Florida, and had a positive point-of-care RT-qPCR test for HRSV between August 2022 and March 2023. Both inpatient and outpatient encounters were included, and clinical and demographic data were collected from the electronic medical record.

### 2.2. Descriptive Analysis of Clinical Presentation

Clinical presentation was summarized according to inpatient versus outpatient status. Among inpatients, severity was assessed by admission to the Pediatric Intensive Care Unit (PICU) versus floor admission, need for oxygen support above low-flow, and length of hospital stay. Because χ^2^ tests for categorical comparisons had low statistical power, except for variables strongly linked to severity (risk ratio > 2.5), only descriptive analyses are presented.

### 2.3. Genome Sequencing

Libraries were prepared using Illumina DNA Prep with Enrichment Respiratory Virus Oligo Panel v2, and sequencing was performed on the Illumina NextSeq 1000 platform (Illumina, Inc., San Diego, CA, USA). Genomes were quality-checked (>Q20) and assembled using VIGEAS (Viral Genome Assembly) workflow (https://github.com/khourious/vigeas, accessed on 30 June 2024). The quality criteria for genome assemblies included >98% identity to the corresponding reference genome and >10 times coveragedepth. Sequencing was attempted for all RT-qPCR-positive samples with available genetic material, and Ct values were not used as an inclusion criterion for sequencing. To avoid the introduction of sequence quality bias, the samples that generated insufficient read numbers or assemblies that did not meet the quality criteria were not included in downstream genomic analyses. Assembly metrics and NCBI GenBank accession numbers are provided in Table S1.

### 2.4. HRSV Genotyping and Phylogenetic Analyses

The Nextclade HRSV tool was used for HRSV-A and HRSV-B genotyping [12]. A dataset of references sequences from GISAID (Global Initiative on Sharing All Influenza Data, Munich, Germany) was then constructed (https://doi.org/10.55876/gis8.250722ng). To reduce geographic overrepresentation, publicly available reference sequences were downsampled using TARDiS to retain representative temporal and geographic diversity across regions. However, because genomic surveillance intensity varies substantially across countries and US states, residual sampling bias may remain [13].

Each dataset was aligned using MAFFT v7.455 [14] and manually edited using AliView v1.26 [15]. Maximum Likelihood (ML) trees were reconstructed in IQ-TREE v1.6.12 [16,17], with branch and node support assessed using 1000 ultrafast bootstrap replicate [17] and SH-like approximate likelihood ratio test [18].

Bayesian phylodynamic analyses were conducted in BEAST v1.10.5pre [19] with BEAGLE v4.0 [20] packages. Following the modeling framework of Langedijk et al. [21], we adopted an uncorrelated lognormal relaxed molecular clock to accommodate rate variation across branches, gamma-distributed site-rate heterogeneity to account for among-site variation in substitution rates, and a Bayesian Skygrid coalescent tree prior to allow flexible reconstruction of effective population size dynamics without imposing a parametric demographic model [22,23]. MCMC analyses were run in duplicate for 100 million iterations, with sampling every 50,000 steps. Effective sample size (ESS) values > 200 were confirmed in Tracer 1.7 [24]. Runs were combined using LogCombiner [19], and 1000 trees per lineage were subsampled.

Phylogeographic analysis was performed using an asymmetric substitution model with Bayesian Stochastic Search Variable Selection [25] to infer global and US transmission routes, which were summarized using Markov jumps and assessed with Bayes Factors. Trees were processed with TreeMarkovJumpHistoryAnalyzer [26], visualized using Circlize [27] and ggtree, and the maximum clade credibility (MCC) tree was generated in TreeAnnotator. Additional methodological details are provided in the Supplementary Materials.

The average time between effective population size (*Ne*) peaks before and after the COVID-19 pandemic bottleneck was estimated using a custom Python 3.10 script, enabling comparison of periodicity changes between periods (see Supplementary Materials for more details).

### 2.5. HRSV Epitope Diversity

To evaluate HRSV epitope entropy and diversity, experimentally tested B-cell and T-cell epitopes from the HRSV G and F proteins were retrieved from the Immune Epitope Database (IEDB; search performed on 2 April 2025—https://www.iedb.org/) using “HRSV” as the organism, “human” as the host, “infectious disease” as the disease category, and positive assays only [28]. Shannon entropy was calculated to measure epitope variability in pre- and post-pandemic HRSV-A and HRSV-B datasets [29]. Differences between periods were tested using the Wilcoxon signed-rank test and Student’s *t*-test as proper. Because multiple epitope comparisons were exploratory, results were interpreted cautiously and considered hypothesis-generating. Details of the IEDB search strategy, epitope selection, entropy calculations, and statistical analyses are provided in Supplementary Materials.

## 3. Results

### 3.1. Patient Characteristics

**Figure 1:**
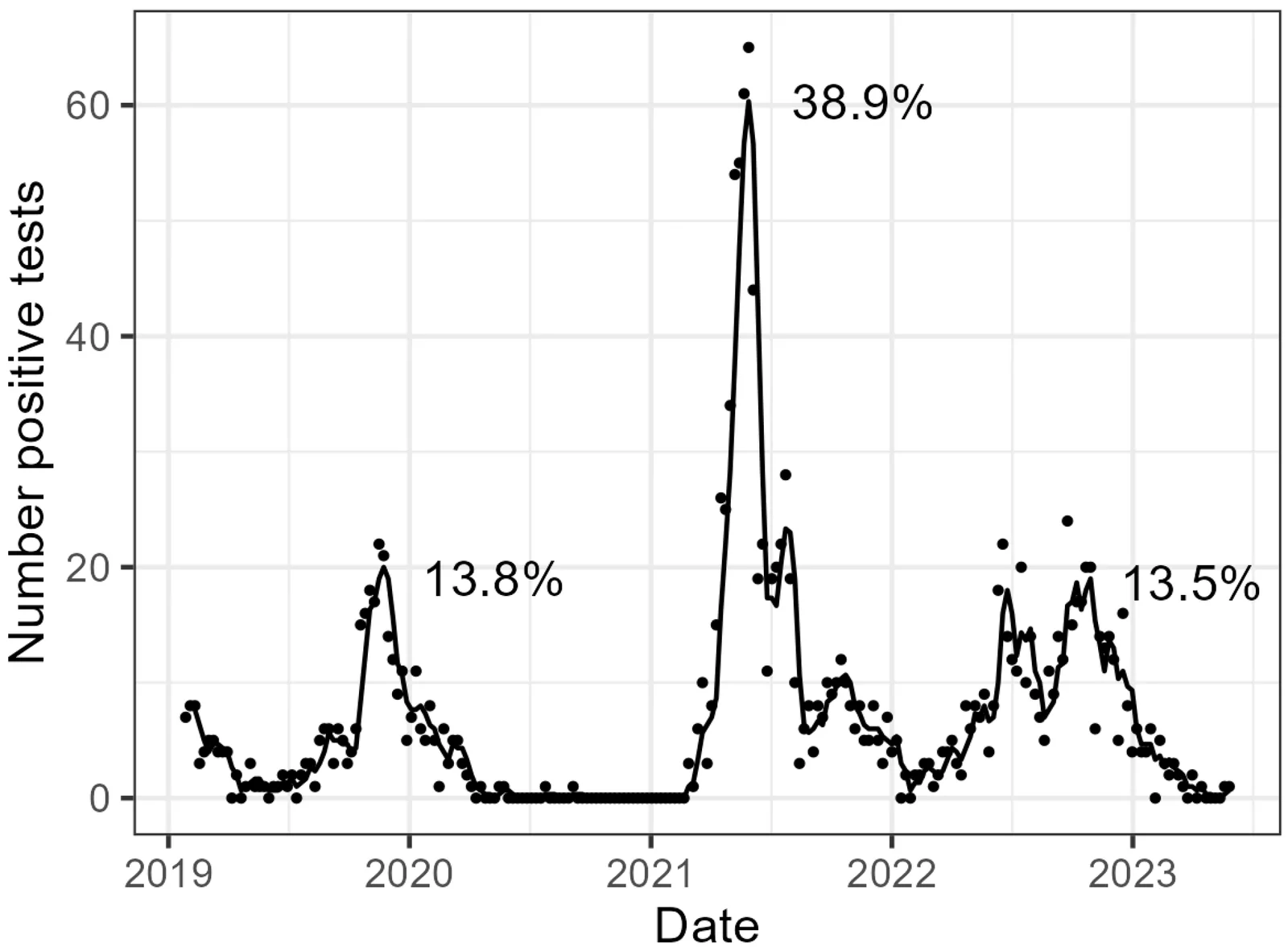
Temporal distribution of HRSV-positive tests at UF Health Shands Hospital. Weekly number of HRSV-positive tests among samples included in syndromic surveillance from 2019 to 2023. Points indicate the observed number of RSV-positive tests at each time point, and the solid line represents the three-week rolling average. Text labels indicate the peak rolling average percent of HRSV-positive tests for the main RSV peaks in 2019, 2021, and 2022.

We observed increased numbers of pediatric HRSV cases at a medical center in Florida in the 2021 to 2023 “post-COVID-19” period (Figure 1). This study evaluated a cohort of 48 pediatric patients, both inpatients and outpatients, aged 0–18 years, collected between August 2022 and March 2023 with symptomatic bronchiolitis, positive RT-qPCR for HRSV, and for whom complete clinical data were available. One patient had two outpatient visits within 30 days; the duplicate was removed, resulting in a final population of n = 47.

Nineteen (40.4%) of the children included had a comorbidity: three neurological; fourteen pulmonary; one cardiac; and one had a neurological and cardiac comorbidity. Twenty-two (73%) of the 30 inpatients required supplemental oxygen therapy above low-flow nasal cannula, and of those, 9 (41%) required positive pressure ventilation. Thirteen (43%) were admitted to the PICU. In the comparison of covariates between inpatient (n = 30) and outpatient (n = 17) children, the inpatient population was younger (median age 203 days vs. 2140 days). Given the small cohort size and the potential influence of age, comorbidities, and hospitalization status on disease outcomes, clinical comparisons were interpreted descriptively. Characteristics of the study cohort are summarized in Table 1.

### 3.2. HRSV Genomes and Lineage Characterization

To identify the Alachua County, Florida HRSV types and genotypes of the strains circulating in the medical center, 32 samples, with available genetic material, of the 48 RT-qPCR HRSV-positive patients underwent whole genome sequencing. Of the 32 sequenced HRSV samples, five samples resulted in a low number of reads, and their genomes could not be assembled. Among the 27 successfully assembled genomes, four were HRSV-B and 23 HRSV-A. Two HRSV samples showed evidence of coinfection, one with Human bocavirus (HBoV1) and one with HBoV1 and Human polyomavirus 3 (KIPyV). To these data, we added three additional HRSV-A sequences from UFHealth (Alachua County, FL, USA) cases for which clinical metadata were unavailable. These sequences were included in the genomic characterization and phylodynamic analyses, resulting in a total of 30 Florida genomes in the analysis (26 HRSV-A and 4 HRSV-B) (Table S1).

**Figure 2:**
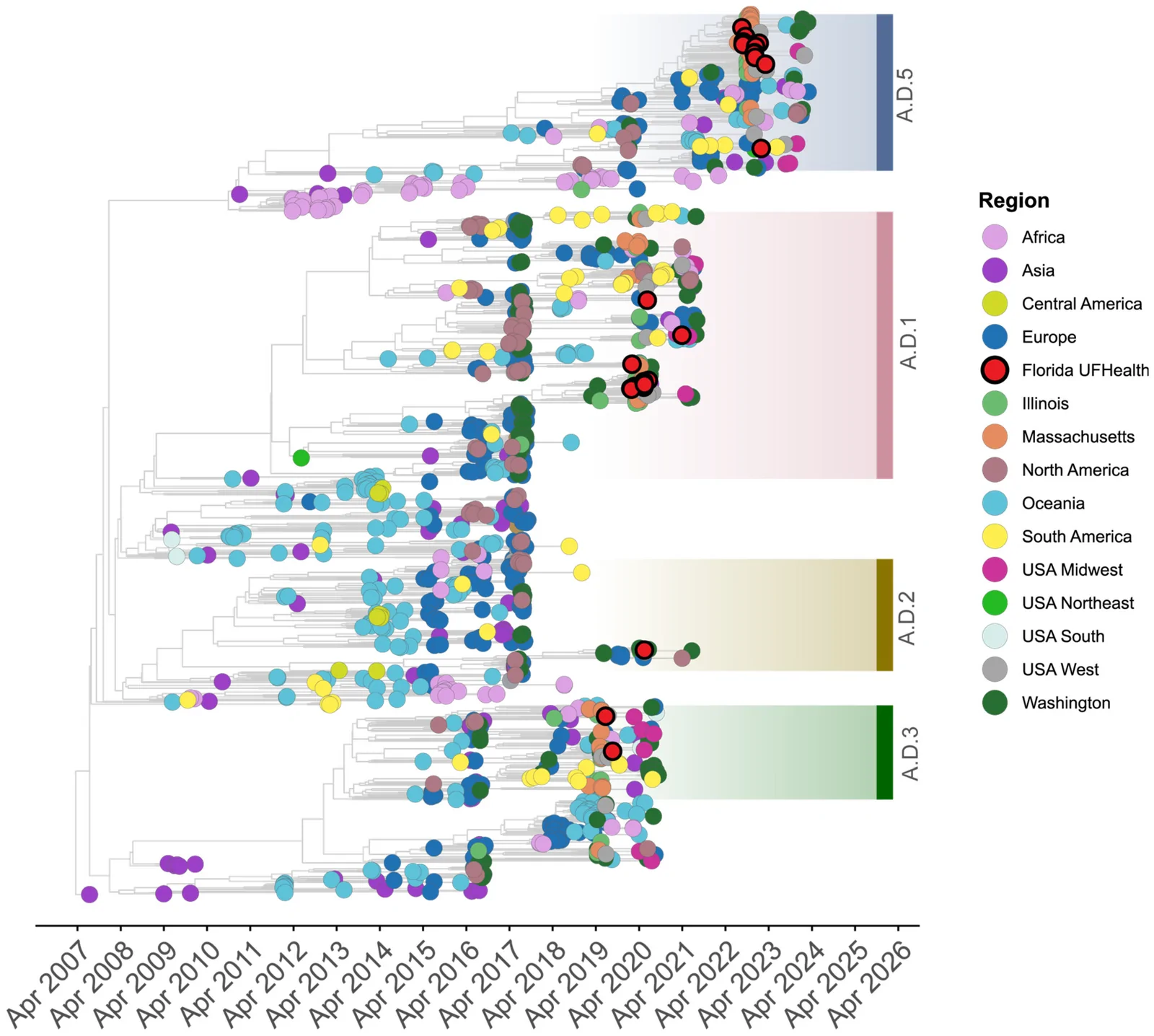
Time-scaled Bayesian phylogenetic reconstruction of HRSV-A whole-genome sequences. The maximum clade credibility tree was inferred from whole-genome sequences of human respiratory syncytial virus type A (HRSV-A). HRSV-A genotype clades are indicated by colored vertical bars on the right side of the tree. Circles at the tree tips represent individual sequences and are colored according to their sampling geographic region, as indicated in the figure color key. The time scale is shown along the x-axis. All Florida sequences originated from UFHealth (Alachua County) and are indicated by orange circles with black outlines.

The Florida HRSV-A genotypes identified using the full genome classification were A.D.1 (n = 11); A.D.2 (n = 1); A.D.3 (n = 2); and A.D.5 (n = 12). All 26 sequences were classified as GA2.3.5 in the G-protein classification. The Florida HRSV-B genotype was B.D.E.1, and GB5.0.5a for the full genome and G-protein classification for all sequences (Figure 2 and Figure S1). These represent the first available HRSV sequences from Alachua County, Florida (UFHealth) and comprise all Florida sequences analyzed in this study. These genomes provide local genomic surveillance data from an underrepresented setting. Nonetheless, the predominance of HRSV-A and detected coinfections should be interpreted cautiously given the limited number of genomes analyzed.

Inpatients were overrepresented among patients with available sequence: 21/27 patients with sequences were inpatients, compared with 9/20 patients without sequences. Among sequenced patients, 17/23 infected with HRSV-A were inpatients and 9/23 were admitted to the ICU, compared to 4/4 and 0/4 patients infected with HRSV-B, respectively. The four HRSV-B inpatients had a median age of 38 days, compared to 391 days among HRSV-A inpatients. Receipt of supplemental oxygen support was similar between types, occurring in 13/17 HRSV-A inpatients (76.5%) and 3/4 HRSV-B inpatients (75%) (Table S1). Within the limits of the available data and considering the overrepresentation of hospitalized patients among sequenced samples, no clear association was observed between Florida sequence clustering or genetic features and available clinical characteristics, including disease severity.

### 3.3. HRSV Phylodynamic Analysis

To gain insights into the genetic diversity and inferred transmission dynamics of HRSV we carried out phylodynamic analyses for both HRSV-A and HRSV-B.

#### 3.3.1. HRSV-A Phylodynamics

For HRSV-A phylodynamics, our initial dataset comprised 1352 HRSV-A sequences, including the 26 Florida UFHealth sequences (all from this study), 370 from different regions of the US, and 956 from other parts of the world. Consistent with previous observations [7,30], US strains from outbreaks in Washington state and Illinois were also distributed in different lineages (A.D.1, A.D.2, A.D.3, and A.D.5), appearing to branch off from European strains, and intermixed with our new Florida isolates.

**Figure 3:**
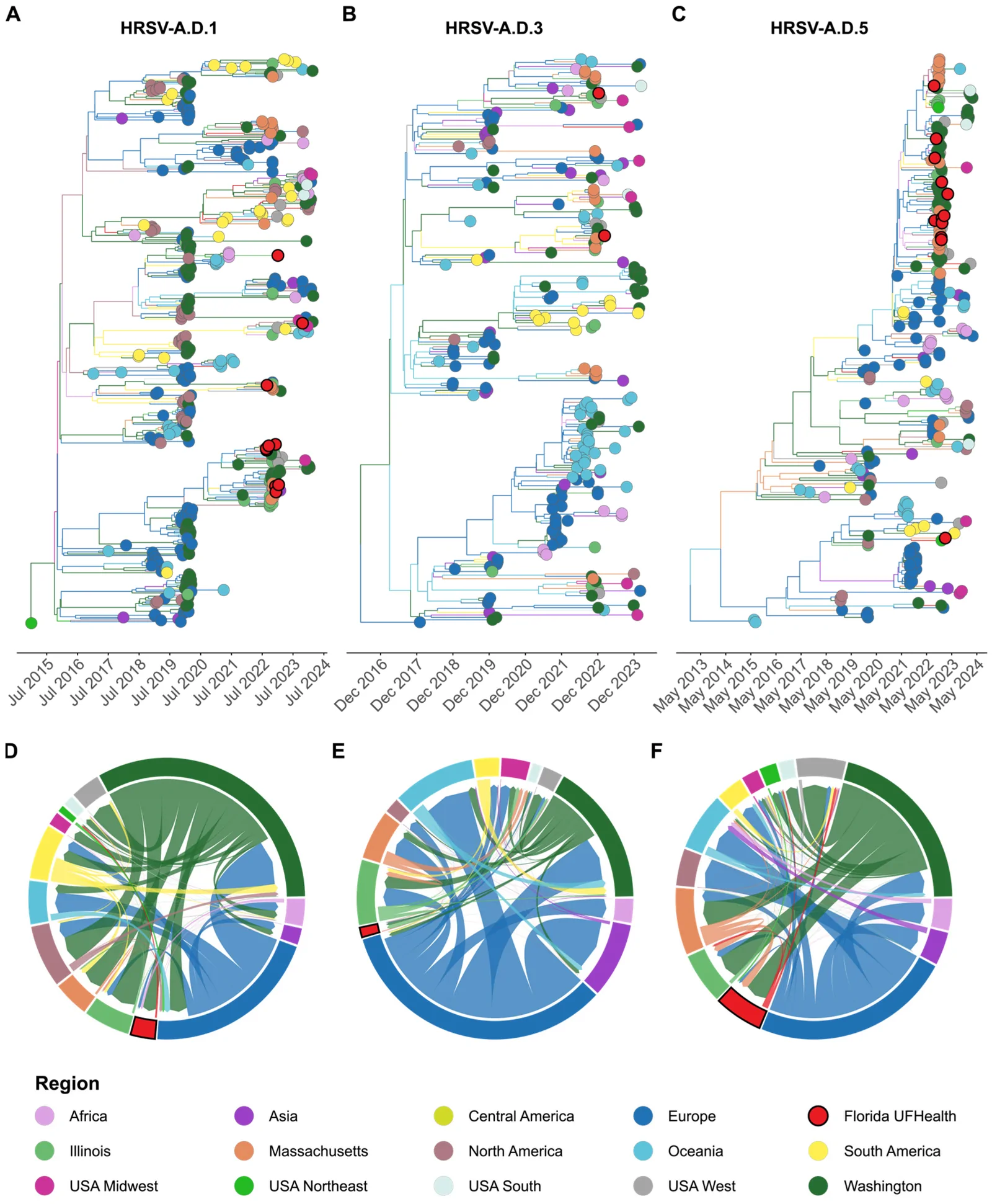
Phylogeographic patterns of HRSV-A lineages detected in Florida. Time-scaled phylogenetic trees are shown for (**A**) HRSV-A.D.1, (**B**) HRSV-A.D.3, and (**C**) HRSV-A.D.5. Tip colors indicate the sampling location or geographic region of each sequence, as assigned in the figure color key. Circular migration plot summarizing inferred viral movements among regions for (**D**) HRSV-A.D.1, (**E**) HRSV-A.D.3, and (**F**) HRSV-A.D.5. Colors indicate geographic assignments, and ribbons represent inferred connections weighted by Markov jumps, which estimate transitions between geographic states. All Florida sequences originated from UFHealth (Alachua County) and are indicated by orange circles with black outlines.

**Figure 4:**
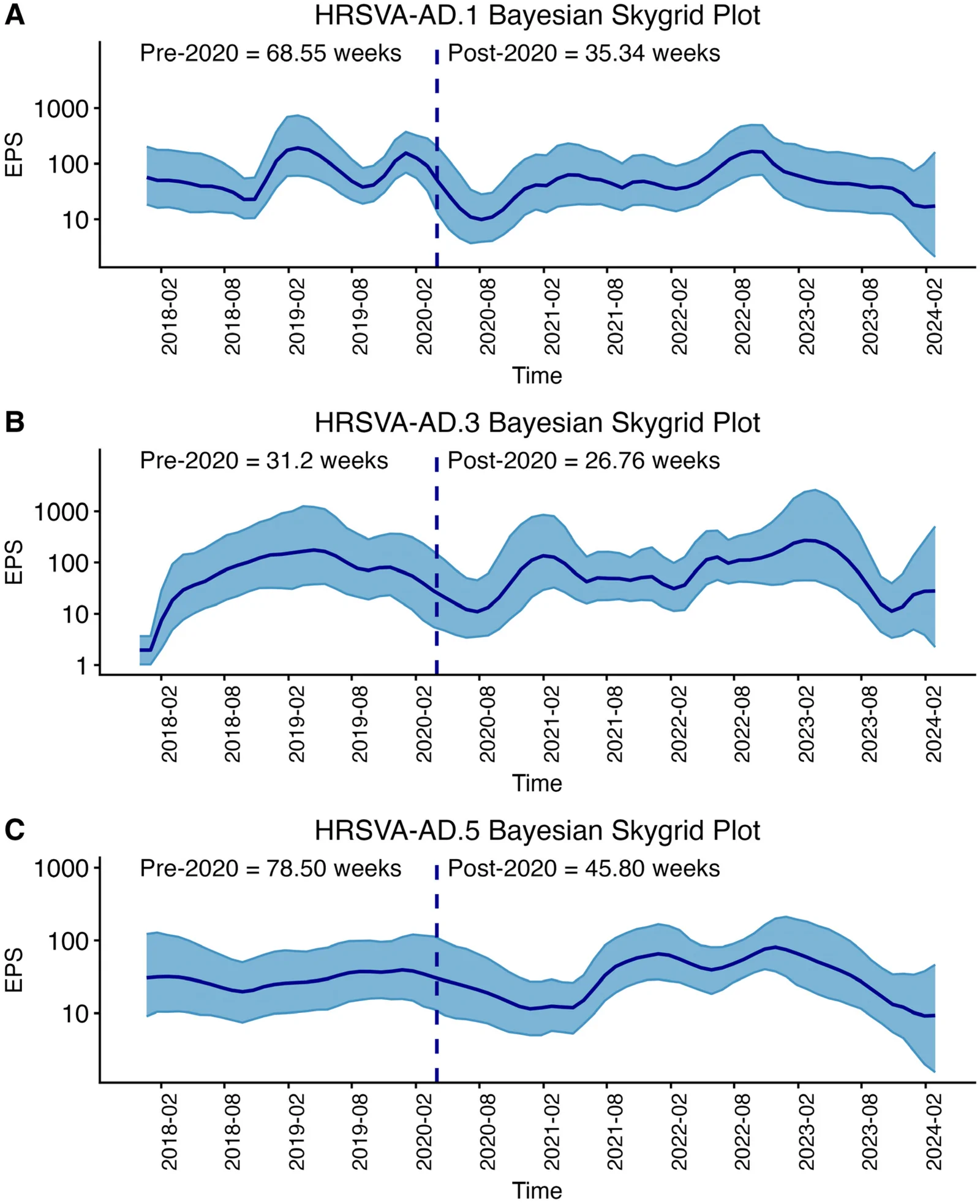
Bayesian Skygrid plots showing changes in relative effective population size over time for HRSV-A clades A.D.1 (**A**), A.D.3 (**B**), and A.D.5 (**C**). The dark blue line represents the median estimate of effective population size, and the light blue shaded area represents the uncertainty interval. The vertical dashed line indicates the onset of the COVID-19 pandemic period used to separate pre- and post-2020 dynamics. Values shown above each panel indicate the estimated average interval between effective population size peaks before and after 2020, highlighting changes in inferred bottleneck periodicity following the pandemic disruption.

Lineages including US strains were analyzed with the Bayesian framework to uncover their phylogeographic and population dynamic patterns (see Supplementary Materials). The inferred Bayesian maximum clade credibility (MCC) tree suggests that, for A.D.1, ancestral nodes compatible with possible first introductions into Florida were dated around mid-October 2021, with a 95% high posterior density interval (95% HPD) of November 2020–March 2022 (Figure 3A). Phylogeographic reconstruction (Figure 3A) may be consistent with a possible pattern of domestic introductions, with Figure 3B perhaps hinting at an early contribution to some Florida infections (Markov Jumps = 5, 95% HPD, interval: 5–7). However, it is important to consider that such a pattern may be reflecting reduced sample size and a sampling bias, given that genomic data remain unavailable for many regions, with an uneven geographical distribution dataset. The Bayesian Skygrid reconstruction (Figure 4A), which estimates relative changes in viral effective population size, *Ne*, suggests sequential population bottlenecks followed by periods of expansion, broadly in line with what is expected for a pathogen associated with seasonal outbreaks. A population bottleneck can be observed in April 2020, coinciding with the onset of the COVID-19 pandemic and the implementation of public health measures such as social distancing and face mask interventions. This was followed by sequential small bottleneck/*Ne* expansion cycles, culminating in a small peak in late 2022. To understand if the average time between peaks changed after the onset of the COVID-19 pandemic, the average peak time interval from one peak to the next before and after the 2020 bottleneck was estimated (Supplementary Materials). This analysis suggests a shorter average interval between *Ne* peaks after 2020 (35.4 weeks, 95% HPD 28.3–35.3) compared to before 2020 (68.55 weeks, 95% HPD 70.7–42.4). However, in the post-2020 period we noted sustained low *Ne* in gradual growth, with a sharp peak in mid-2022, making earlier post-2020 peaks less apparent.

Similar patterns were observed for lineage A.D.3 and lineage A.D.5 (Figure 3). A.D.3 phylogeography analysis suggested multiple introductions to the US in the pre-COVID period from Europe (Figure 3C,D) and a decrease in *Ne* coinciding with the onset of the COVID-19 pandemic (Figure 4B). Interestingly, A.D.3 shows a shorter *Ne* periodicity (31.3 weeks, 95% HPD 35.7–24.5) before 2020 compared to A.D.1. However, following the same trend, the interval between *Ne* peaks is even shorter after 2020 (26.8 weeks, 95% HPD 27.9–36.8), with a consistent peak appearing in late 2022. For A.D.5, the analyses are compatible with an initial introduction from Europe prior to 2020, with subsequent viral migration patterns within the US suggesting that transmission was potentially sustained largely through national dissemination. For Florida specifically, the data are consistent with a domestic pattern, with viral movements occurring during the post-COVID-19 period (Washington state, MJ = 5, 95% HPD interval: 4–6; Massachusetts, MJ = 2, 95% HPD interval: 1–3) (Figure 3E). As seen in A.D.1, this pattern may also be influenced by sampling biases, given the lack of sequence data from several states. The Skygrid reconstruction for A.D.5 (Figure 4C) also showed sequential viral *Ne* bottlenecks followed by *Ne* expansions, with an incline in March 2020 and a shorter time difference between *Ne* peaks after 2020 (45.8 weeks 95% HPD 47.9–43.6) when compared to before 2020 (74.2 weeks 95% HPD 39.3–78.5). The only exception is HRSV-A A.D.2, which does show sequential *Ne* bottlenecks before 2020 (61.4 weeks, 95% HPD 46.1–35.9), followed by a decline during the onset of the COVID-19 pandemic and a subsequent expansion after 2020 (39.5 weeks, 95% HPD 43.9–29.6) (Figure S2).

#### 3.3.2. HRSV-B Phylodynamics

**Figure 5:**
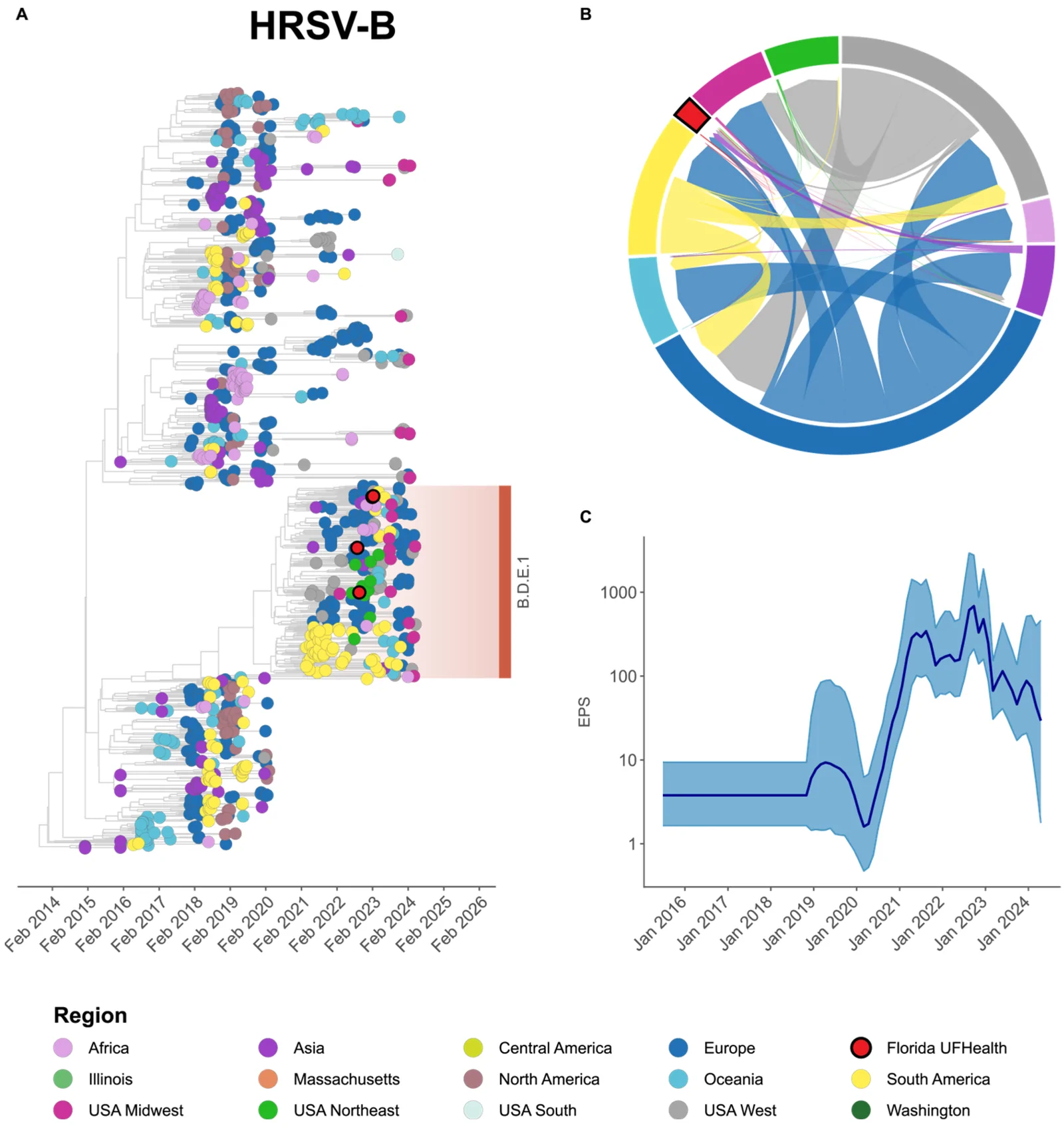
Phylogeographic reconstruction and Bayesian Skygrid analysis of HRSV-B. (**A**) Time-scaled MCC tree of HRSV-B sequences, with branches and tips color-coded according to sampled or inferred geographic region as indicated in the figure color key. The HRSV-B B.D.E.1 clade is highlighted. (**B**) Circular migration plot summarizing inferred viral movements among regions. Colors indicate geographic assignments, and ribbons represent inferred connections weighted by Markov jumps, which estimate transitions between geographic states. (**C**) Bayesian Skygrid plot showing temporal changes in relative effective population size, with the dark blue line indicating the median estimate and the light blue shaded region representing the uncertainty interval. All Florida sequences originated from UFHealth (Alachua County) and are indicated by orange circles with black outlines.

Given the limited number of newly generated local HRSV-B sequences (n = 4), phylogeographic reconstruction tentatively suggested possible viral movements within the US toward Florida around July 2021 (95% HPD: August 2020–August 2022) (Figure 5B). These inferred patterns carry considerable uncertainty and should be interpreted with caution, particularly given the reduced new local sequences and uneven genome sequencing efforts combined with downsampling strategies applied across regions, which may have substantially influenced the reconstructed dynamics. Similarly to HRSV-A, the analysis suggested likely source from Europe to the US (Florida, Midwest, and West regions) around April 2020 (95% HPD: March 2020–July 2020) (Figure 5C).

The Skygrid plot shows an increase in viral *Ne* starting in late 2018, coinciding with the early introduction of HRSV-B from Europe to the US, followed by a dip at the beginning of the COVID-19 pandemic. Viral *Ne* dynamics after the bottleneck at the onset of COVID-19 are similar to observed for HRSV-A, showing sequential *Ne* increases followed by population bottlenecks with 30.5 weeks (95% HPD 24.2–30.5) average frequency.

A probable *Ne* peak around July–August 2021 appears to correspond with the period of NPI relaxation and resumption of population mobility, which may have contributed to increased transmission opportunities during this period, although *Ne* does not directly measure transmission intensity.

### 3.4. Diversity of HRSV Epitopes

First, complete genome amino-acid sequence entropy differences between the pre- and post-pandemic periods were calculated. Mean amino acid entropy was slightly higher in post-pandemic than in pre-pandemic sequences (0.0237 vs. 0.0209; observed difference = 0.0029), however, this difference was not statistically significant (permutation test, *p* = 0.14, 95% bootstrap CI: −0.001 to 0.0067), indicating no detectable genome-wide change in amino acid diversity between periods.

**Figure 6:**
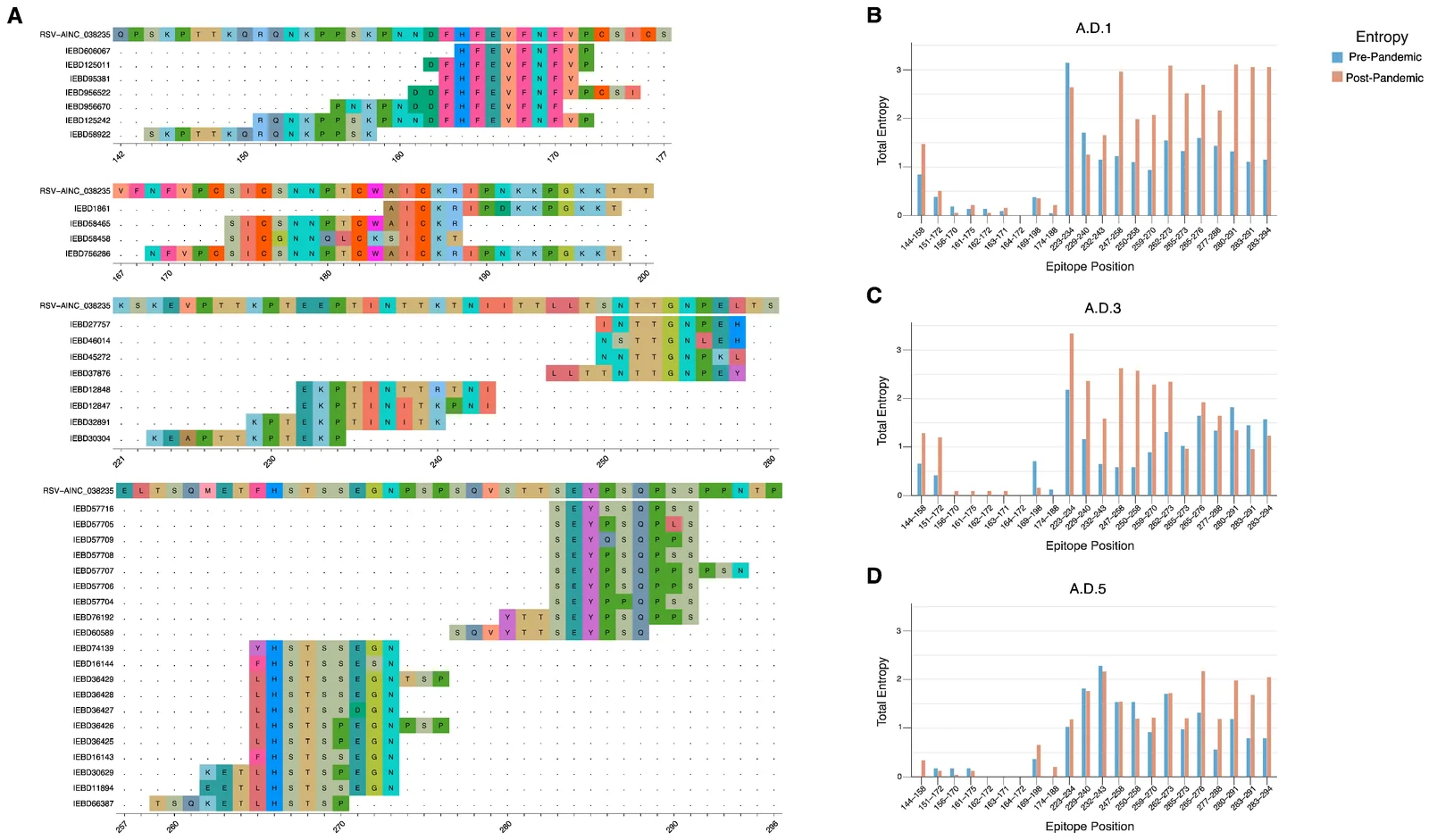
Entropy of HRSV-A G-protein epitopes before and after the COVID-19 pandemic. (**A**) Map of experimentally described G-protein epitopes analyzed in this study, with epitope positions numbered according to the HRSV-A reference sequence NC_038235. (**B**–**D**) Total epitope entropy for HRSV-A lineages circulating in the United States before and after the onset of the COVID-19 pandemic: A.D.1 (**B**), A.D.3 (**C**), and A.D.5 (**D**). Blue bars represent pre-pandemic datasets, and red bars represent post-pandemic datasets. The x-axis indicates the start and end positions of each epitope according to the HRSV-A reference sequence, and the y-axis shows total entropy, with higher values indicating greater amino acid variability within the corresponding epitope region.

Genetic diversity within F- and G-protein epitope regions was then evaluated for each lineage. The three HRSV-A lineages, A.D.1, A.D.3, and A.D.5, showed increased G-protein epitope entropy in the post-pandemic period (Wilcoxon signed-rank test for paired samples: A.D.1 *p* = 0.0008; A.D.3 *p* = 0.008; A.D.5 *p* = 0.013). A similar pattern was observed at non-epitope G-protein sites, with increased post-pandemic entropy in A.D.1, A.D.3 and A.D.5, whereas A.D.2 and HRSV-B showed higher entropy in the pre-pandemic period. Although the G protein is not the primary target of currently approved vaccines, these findings indicate increased amino acid variability within known G-protein epitope regions after the onset of the COVID-19 pandemic for several HRSV-A lineages (Figure 6) [31]. Only HRSV-A A.D.2 and HRSV-B showed a trend toward greater pre-pandemic epitope diversity, although these differences were not statistically significant (*p* > 0.1) (Figure S3).

F-protein epitopes were generally more conserved, as expected given that this region is a major target for vaccine and monoclonal antibody development [31]. Despite this overall conservation, comparison of F-protein epitope entropy distributions before and after the COVID-19 pandemic showed statistically supported differences for A.D.2 (*p* < 0.0001) and A.D.5 (*p* < 0.0288) (Mann–Whitney U test) (Figure S4). Because no significant increase in genome-wide amino acid diversity was detected, the observed changes in epitope-region entropy may reflect localized amino acid variation in antigenically relevant regions. However, these genomic patterns do not directly demonstrate altered antigenicity, immune escape, reduced vaccine effectiveness, or adaptive viral evolution, and functional or immunological studies would be required to determine their biological significance.

## 4. Discussion

The epidemiologic pattern of a decline in pediatric HRSV cases during the COVID-19 pandemic, followed by a resurgence of cases in 2022/2023, has been reported in multiple studies, including studies in the US (Massachusetts [6]; Chicago [7]; Washington state [11]) and internationally (Europe [32]; France [33], China [34], Turkey [35]). Many studies across North America and Europe have also reported an increase in HRSV-related hospitalizations between pre- and post- epidemic time points, with older children in the post-epidemic period and fewer co-morbidities [5,36,37,38,39]. Although an increase in the use of advanced respiratory support was observed here, there were no consistent rises in clinical severity reported across all the different populations [5,36,37,38,39]. Furthermore, two of our HRSV-positive samples represented coinfections with a second respiratory virus. These did not appear to affect the clinical course of the patients, in keeping with prior studies suggesting that HRSV coinfections with other respiratory viruses generally do not affect clinical outcomes [40].

Based on complete genome classification, HRSV-A and HRSV-B are currently divided into two main lineages, A.x and A.D.x for HRSV-A and B.x B.D.x for HRSV-B, further subdivided into 24 HRSV-A and 16 HRSV-B sub-lineages [30]. Among the study isolates, HRSV-A genotypes were diverse (A.D.1, A.D.2, A.D.3, and A.D.5), with all 26 sequences classified as GA2.3.5 based on G protein analysis [41,42]. The four HRSV-B genomes were all B.D.E.1, and GB5.0.5a for the G protein classification. These findings are consistent with studies from multiple regions in the US and globally, which show polyphyletic clustering among HRSV-A, a pattern similar to that observed prior to the COVID-19 pandemic. In contrast, HRSV-B showed monophyletic clustering across geographic regions, suggesting a population bottleneck likely as a result of the COVID-19 intervention measures [7,43]. However, our study population was not adequately powered to evaluate if any genotype could be associated with disease severity, similar to previous studies [11].

Prior to the COVID-19 pandemic, HRSV-A and HRSV-B typically co-circulated with similar prevalence. However, post-pandemic analyses showed a shift toward HRSV-A predominance following the implementation of community-based NPIs [44,45]. For HRSV-A, integration of new Alachua County, Florida genomes with a globally representative dataset suggested that recent US outbreaks were not driven by the emergence of novel lineages, but rather by the re-emergence and continued circulation of previously established clades, particularly A.D.1, A.D.2, A.D.3, and A.D.5. In lineages A.D.1, A.D.3, and A.D.5, the post-pandemic period was also associated with increased G-protein epitope entropy, indicating greater amino acid variability. This HRSV-A genetic diversity has been reported in Australia [45] and Washington state [6]. In contrast, post-pandemic sequences from New Zealand were less diverse, cluttering on a monophyletic clade, suggestive of reduced viral diversity and limited lineage reintroduction [43].

These HRSV-A lineages also showed increased diversity, potentially fueled by domestic viral dissemination. This pattern of domestic virus movements should be interpreted with caution, as it may reflect biases introduced by heterogeneous genomic surveillance programs across the states and limited data availability. These disparities could be tackled on the infrastructure side through more evenly distributed sequencing capacity across state and regional public-health laboratories, together with federated and openly accessible sequence-data repositories. Embedding such standards in national genomic-surveillance frameworks would allow future studies to explicitly account for, rather than simply acknowledge, the heterogeneity of the underlying sequencing infrastructure.

The subsequent lifting of restrictions led to a notable resurgence of HRSV infections [9,46]. Our demographic reconstructions suggested fluctuation in effective population size for HRSV-A and HRSV-B, consistent with continued viral circulation during and after the period of reduced detection. These findings support the interpretation that the substantial post-intervention upsurge was a multifactorial origin, not solely attributable to ‘immunity debt’ from reduced pathogen circulation. Instead, the resurgence may have reflected a combination of a larger susceptible pediatric pool, the rapid resumption of social mixing and school reopenings, the re-establishment of global transmission networks via increased air mobility, and enhanced clinical surveillance that amplified case detection. These immunological, behavioral, ecological, and surveillance-related factors are consistent with existing literature [21,46].

This post-pandemic shift in *Ne* periodicity and increased G protein diversity suggest an accumulation of mutations within known epitopes, which possibly may affect transmission dynamics. In contrast with HRSV-A, HRSV-B presents lower genomic diversity, evidenced by the lower G protein epitope entropy in the post-pandemic cases. US and global HRSV-B isolates belonged to the same lineage, with most sequences clustering within a monophyletic clade, consistent with a previous study [7]. Preliminary phylogeographic analyses of the US sub-clade suggest patterns similar to those observed in HRSV-A, including potential early introductions from Europe and subsequent domestic dissemination. However, because only four newly generated Florida genomes belonged to HRSV-B, inferences regarding HRSV-B introduction times and domestic transmission routes should be considered preliminary and highly provisional. Although downsampling was applied to reduce geographic overrepresentation and improve temporal and spatial balance in the reference dataset, uneven genomic surveillance across countries and US states may still have influenced the reconstructed phylogeographic patterns. While the post-pandemic HRSV-B epidemic showed fluctuations in *Ne* that could reflect variable transmission patterns, these observations require validation with expanded and more geographically representative sampling.

Although complete genome coding regions did not show a significant increase in amino acid diversity after the onset of the COVID-19 pandemic, epitope-level analyses suggested localized variation in antigenically relevant regions. The F protein, a relatively conserved target for RSV vaccine and monoclonal antibody development, showed statistically supported differences in epitope entropy for HRSV-A lineages A.D.2 and A.D.5. These findings should be interpreted as genomic variation within epitope regions, rather than direct evidence of altered antigenicity, immune escape, or adaptive evolution. Given the recent introduction of maternal RSVpreF vaccination and long-acting monoclonal antibodies such as nirsevimab in the United States during 2023, these data, combined with additional data, will be beneficial for establishing a pre-rollout or early-rollout genomic baseline for future surveillance of antigenically relevant F- and G-protein regions [47,48].

Together, our findings illustrate how pandemic-associated changes in population behavior and public health interventions may have reshaped HRSV transmission dynamics in the post-pandemic period. Our results suggest that, in the aftermath of the COVID-19 pandemic, the virus may have undergone genetic changes, potentially associated with the accumulation of amino acid variation within known epitope regions. Future studies with functional and immunological validation will be valuable to better understand the impact of F- and G-protein diversification on measurable biological effects on the HRSV immune response landscape and target treatments [49].

Therefore, the results underscore the critical role of robust and continuous genomic surveillance systems in monitoring viral evolution, identifying emerging lineages, and informing timely public health responses. Although this study incorporates valuable recent data, including the first HRSV genome sequences from Alachua County, Florida, a few limitations must be highlighted. Uneven geographic coverage across US states may limit the resolution of inferred transmission pathways, particularly for HRSV-B, for which limited post-pandemic sequences were available. To improve HRSV surveillance and mitigate its spread in the United States, standardized state-level sampling metrics, harmonized metadata collection, and coordinated national genomic surveillance infrastructure would be beneficial for reducing geographic sampling disparities and improving the reliability of inferred domestic transmission pathways. Although subsampling strategies minimize bias in the database of available sequences, residual sampling bias may persist due to uneven sequencing efforts and data availability across regions and time. This limitation should be considered when interpreting phylogeographic patterns, which may partly reflect these biases rather than universal trends. In addition, the small sample size precluded adequately powered statistical analysis of demographic and viral factors associated with severity in this post-pandemic population.

Nonetheless, our results illustrate changes in post-pandemic HRSV population genetics, inferred population dynamics, and epitope-region variability, supporting the need for continued genomic surveillance. Sustained and expanded genomic surveillance programs at the regional level may contribute valuable epidemiological intelligence for early variant detection and support the development of evidence-informed public health interventions during future HRSV transmission seasons.

## Figures and Tables

**Table 1:** Characteristics of inpatient and outpatient cohorts stratified by age.

	Overall (N = 47)	0–29 Days (N = 4)	30 Days to 6 Months (N = 12)	6+ Months (N = 31)
**Sex**				
Female	21 (44.7%)	2 (50.0%)	5 (41.7%)	14 (45.2%)
Male	26 (55.3%)	2 (50.0%)	7 (58.3%)	17 (54.8%)
**Gestational age at birth**				
Full-term	38 (80.9%)	3 (75.0%)	10 (83.3%)	25 (80.6%)
Pre-term before 37 weeks	8 (17.0%)	1 (25.0%)	2 (16.7%)	5 (16.1%)
**Underlying Comorbidities ^†^**				
None	28 (59.6%)	4 (100%)	9 (75.0%)	15 (48.4%)
At least one	19 (40.4%)	0 (0%)	3 (25.0%)	16 (51.6%)
**Admission Unit**				
Outpatient	17 (36.2%)	0 (0%)	2 (16.7%)	15 (48.4%)
Floor	17 (36.2%)	3 (75.0%)	4 (33.3%)	10 (32.3%)
ICU	13 (27.7%)	1 (25.0%)	6 (50.0%)	6 (19.4%)
Oxygenation Support above Low Flow Nasal Cannula *				
None	8 (26.7%)	1 (25.0%)	1 (10.0%)	6 (37.5%)
High Flow Nasal Cannula	13 (43.3%)	1 (25.0%)	6 (60.0%)	6 (37.5%)
Non-invasive Positive Pressure Ventilation	8 (26.7%)	2 (50.0%)	3 (30.0%)	3 (18.8%)
Invasive Ventilation (Conventional, HFOV)	1 (3.3%)	0 (0%)	0 (0%)	1 (6.3%)
Required Escalation of Support *				
No	8 (26.7%)	1 (25.0%)	1 (10.0%)	6 (37.5%)
Yes	22 (73.3%)	3 (75.0%)	9 (90.0%)	10 (62.5%)
Length of Stay *				
Median [Range]	2.00 [0, 11.0]	5.50 [3.00, 11.0]	2.00 [0, 6.00]	2.00 [1.00, 10.0]

^†^ Comorbid conditions include neurological, cardiac, and pulmonary comorbidities. * Among inpatients (n = 30).

## Data Availability

All sequences were submitted to NCBI GenBank database, and accession numbers can be found on Table S1. All genome sequences and associated metadata in the collected from GISAID used in the dataset from this study are published in GISAID’s EpiRSV database. To view the contributors of each individual sequence with details such as accession number, Virus name, Collection date, Originating Lab and Submitting Lab and the list of Authors, visit EPI_SET_250722ng. DOI: https://doi.org/10.55876/gis8.250722ng. All the BEAST xmls, log files and python scripts can be found in GitHub (https://github.com/emanuelegustani/Increased_genomic_diversification_frequency_HRSV_Aand_B_in_a_pediatric_cohort_in_the_United-States).

## Supplementary Materials

The following supporting information can be downloaded at: https://www.mdpi.com/article/10.3390/microorganisms14081656/s1, Figure S1. Nextclade HRSV clade classification for Florida sequences. Nextclade classification of Florida HRSV genomes showing (A) HRSV-A and (B) HRSV-B clade assignments. HRSV-A sequences were assigned to clades A.D.1 [green], A.D.2 [light green], A.D.3 [yellow], A.D.5 [orange], and A.D.5.2 [red], whereas all HRSV-B sequences were assigned to B.D.E.1 [light orange]. Colors denote Nextclade-defined clade membership; Figure S2. Phylogeographic reconstruction and Bayesian Skygrid analysis of HRSV-A Clade A.D.2. (A) Time-scaled MCC tree of HRSV-A Clade A.D.2 sequences, with branches and tips color-coded according to sampled or inferred geographic region as indicated in the figure color key. All Florida sequences originated from UFHealth (Alachua County) and are indicated by orange circles with black outlines. (B) Circular migration plot summarizing inferred viral movements among regions. Colors indicate geographic assignments, and ribbons represent inferred connections weighted by Markov jumps, which estimate transitions between geographic states. (C) Bayesian Skygrid plot showing temporal changes in relative effective population size, with the dark blue line indicating the median estimate and the light blue shaded region representing the uncertainty interval; Figure S3. Entropy of HRSV-A G-protein epitopes before and after the COVID-19 pandemic. Total epitope entropy for HRSV-A A.D.2 (A) and HRSV-B (B) circulating in the United States before and after the onset of the COVID-19 pandemic. Blue bars represent pre-pandemic datasets, and red bars represent post-pandemic datasets. The x-axis indicates the start and end positions of each epitope according to the reference sequence NC_038235 (HRSV-A) and NC_001781 (HRSV-B), and the y-axis shows total entropy, with higher values indicating greater amino acid variability within the corresponding epitope region; Figure S4. Dot plots with overlaid bars show entropy values (y-axis) calculated for four HRSV-A lineages (A.D.1, A.D.2, A.D.3, and A.D.5) from genomes before (pre-pandemic) and after (post-pandemic) the COVID-19 pandemic period. Each point represents an individual protein F epitope, with bars indicating central tendency and dispersion for each group. Statistical comparisons between pre-pandemic and post-pandemic groups for each lineage were performed using a non-parametric Mann–Whitney U test. Exact *p*-values are shown above each panel. Entropy is used as a measure of within-host viral genetic diversity, with higher values indicating increased sequence heterogeneity within the sampled viral population; Table S1. HRSV sequences assembly metrics and genotype information. References [12,13,14,15,16,17,18,19,20,22,23,24,25,26,27,28,29,50,51,52,53,54,55,56,57,58,59,60,61,62,63,64] are cited in the supplementary materials.

## Abbreviations

The following abbreviations are used in this manuscript:

HRSV	Human Respiratory Syncytial Virus
US	United States
NPIs	Non-pharmaceutical Interventions
RT-qPCR	Reverse Transcription-quantitative Polymerase Chain Reaction
PICU	Pediatric Intensive Care Unit
COVID-19	Coronavirus disease 2019
HBoV1	Human bocavirus
KIPyV	Human polyomavirus 3
MCC	Bayesian maximum clade credibility
HPD	Highest Posterior Density
