# Supplementary material for: Genomic Diversity of Human Respiratory Syncytial Virus (HRSV) Type A in a Pediatric Cohort in the United States After the Onset of the COVID-19 Pandemic
Source: Microorganisms. 2026 Jul 29;14(8):1656. doi: 10.3390/microorganisms14081656 (PMC13515401; doi:10.3390/microorganisms14081656)
Supplement: Supplementary file 1 [file microorganisms-14-01656-s001.zip › Supplementary Materials.pdf]

Supplementary Materials

Additional Methods

Study design and population

This is an IRB-approved (IRB202302154), retrospective population-based cohort study. The study population consists of 48 pediatric patients aged 0-18 years who were symptomatic with HRSV bronchiolitis who were seen at UFHealth Children's Hospital at the University of Florida Health System, Alachua County, that had a positive RT-qPCR for HRSV between August 2022 and March 2023. Both inpatient and outpatient encounters were included, which varied amongst general pediatric primary care offices, the Pediatric Emergency Department, the Pediatric Inpatient Hospital floor, and the Pediatric Intensive Care Unit (PICU). Thirty-two of the 48 positive RT-qPCR samples underwent whole genome sequencing.

Data and sample collection

Clinical and demographic data were accessed securely through the Electronic Medical Record and were manually collected through chart review. Nasopharyngeal swab samples were collected and analyzed using point-of-care RT-qPCR testing.

Descriptive analysis of clinical presentation

We summarized the clinical presentation of patients by whether they were inpatient or outpatient, and three markers of severity within inpatients: admission to the Pediatric Intensive Care Unit (PICU) vs. admission to the pediatric floor; oxygenation support above low-flow nasal cannula (high-flow nasal cannula, non-invasive positive pressure ventilation, and invasive ventilation including conventional and high-frequency oscillatory ventilation, HFOV); and length of stay in the hospital. We reported, by age group, the proportion of these severe outcomes, along with demographic and other clinical characteristics (specifically sex, gestational age at birth, and the presence of any comorbidities including neurological, cardiac, and pulmonary). We conducted power analyses for χ^2 tests comparing the distribution of categorical variables between severe and non-severe cases. Except in cases where a variable was strongly associated with a severity outcome (e.g. with an associated risk ratio greater than 2.5), χ^2 tests had low power. Therefore, we present only a descriptive analysis of clinical data.

Genome Sequencing

A total of 32 HRSV positive samples were sequenced. RNA was extracted using QIAamp Viral RNA Kit (Qiagen Inc., Valencia, CA, USA, Cat. No. 52906). The libraries were then prepared using the Illumina DNA Prep with Enrichment with Respiratory Virus Oligo Panel (RVOP) v2 (Illumina, San Diego, CA, USA). All libraries were pooled together in equimolar amounts prior to sequencing. The fragment length distribution was assessed using the High Sensitivity DNA ScreenTape Kit (Agilent Technologies, Santa Clara, CA, USA) on the Agilent 4150 TapeStation system (Agilent Technologies, Santa Clara, CA, USA) and the concentration was assessed using Qubit 1X dsDNA High Sensitivity Assay Kit (Thermo Fisher Scientific, Waltham, MA, USA, Cat. No. Q33230 or Q33231) on the Qubit 4 Fluorometer (Thermo Fisher Scientific, Waltham, MA, USA, Cat. No. Q33216). The library pool was denatured and diluted to a final loading concentration of 1000 pM with 4% PhiX spike-in and then sequenced on an Illumina NextSeq 1000 (Illumina, San Diego, CA, USA, Cat. No. SY-410-1003) using a 300-cycle NextSeq 1000/2000 P1 Reagent Kit v2 (Illumina, San Diego, CA, USA, Cat. No. MS-102-2002) to generate 150 bp paired-end reads. All protocols followed the manufacturer's instructions.

Genome assembly

The FASTQ files were trimmed using fastp v0.23.2[50] to remove low-quality base pairs. A pre-assembly was performed using the metaviralSPAdes implemented in SPAdes v4.0.0[51]. The assembly contigs were then input for BLASTn[52] against a custom-built reference database developed based on the RVOP enrichment panel. The database also included Human Influenza Vaccine Composition available on GISAID (https://gisaid.org/resources/human-influenza-vaccine-composition). The assembly was performed by Burrows-Wheeler Aligner (BWA) v0.7.17[53] with the reference genomes with an identity >98%. The variant calling and consensus sequence generation were performed using iVAR v1.3.1[54]. The consensus sequences were masked with "N" at regions with coverage depth <10. The assembly statistics were calculated with SAMtools v1.16.1 (using HTSlib v1.16.1)[55] and Seqtk v1.3-r106 (https://github.com/lh3/seqtk). This entire workflow is available at https://github.com/khourious/vigeas , accessed on 30th June 2024. All sequences were submitted to NCBI GenBank database. Sequencing was attempted for all RT-qPCR-positive samples with available genetic material; Ct values were not used as an inclusion criterion for sequencing. Samples that generated insufficient read numbers or assemblies that did not meet the quality criteria were not included in downstream genomic analyses. Assembly metrics and accession numbers can be found on Table S1.

Sanger sequencing

Three PCR positive samples were sequenced using Sanger sequencing. For this, virus RNA was extracted and purified directly from swab specimens or virus isolated in Vero E6 cells using a QIAamp Viral RNA Mini Kit (Qiagen Inc., Valencia, CA, USA). First-strand synthesis was performed using random 9-mers and an Accuscript High Fidelity 1st strand cDNA kit (Agilent Technologies, Santa Clara, CA, USA)[56]. PCR was performed using Q5 DNA polymerase (New England BioLabs, Ipswich, MA, USA) and the primers described by M.E. Bose et al.[57] to produce tiled short-amplicons with an average length of 650 bp. The amplicons were purified from agarose gels prior to sequencing. The 5′ and 3′ ends of HRSV-A genomes were determined using a Rapid Amplification of cDNA Ends (RACE) kit (Life Technologies, Inc., Carlsbad, CA, USA). The sequencing reads were trimmed to eliminate amplicon primers and low-quality sequences, and the virus sequence assembled manually.

HRSV genotype identification

To identify the HRSV-A and HRSV-B genotype, a multiple alignment of sequences for each HRSV type was performed using MAFFT (Multiple Alignment using Fast Fourier Transform) v7.455 [14]. Subsequently, the alignments were assigned using Nextclade V.3.10.0 HRSV tool for genotyping[12]. We used Aliview v.1.26[15] to inspect, visualize and edit the alignments.

Phylogenetic analyses

To understand the HRSV dynamics in a global context, we construct a background dataset downloading genomes from GISAID that were complete and had information regarding collection date and location (https://doi.org/10.55876/gis8.250722ng). To reduce the computational burden and sampling bias in subsequent analyses, we applied a down-sampling strategy to the databases using TARDiS, which uses a genetic algorithm to minimize bias by selecting sequences within a sampling from different geographic regions and timepoints[13]. In particular, TARDiS uses a genetic algorithm to minimize sampling bias by selecting sequences from different geographic regions and timepoints that match the database underlying genetic diversity distribution, thus assuring that both highly similar and highly divergent sequences are adequately represented in the down-sample. For a more cohesive dataset, we down-sampled only sequences from locations and timepoints that were overrepresented, not losing diversity and representation. The down-sampling was done by states for the US sequences and by country for the rest of the world. The final dataset consisted of 1352 genomes for HRSV-A (Africa = 109, Asia = 94, Europe = 357,

Oceania = 226, Central America = 11, North America = 95, South America = 64, USA = 370, and Florida = 26) and 1084 genomes for HRSV-B (Africa = 77, Asia = 136, Europe = 433, Oceania = 103, North America = 72, South America = 140, USA = 119, and Florida = 4).

For the Maximum Likelihood (ML) tree reconstruction, sequences were aligned using MAFFT v7.455[9] and edited using AliView v1.26[15]. The ML trees were constructed in IQ-TREE v1.6.12[14,17] using the best fitted model of evolution chosen according to Bayesian Information Criterion by the ModelFinder[58] (GTR+F+I+R5 for HRSV-A and GTR+F+I+R6 for HRSV-B) implemented in IQ-TREE. Statistical support for nodes was assessed with a 1000-replicate ultrafast bootstrap[17], and the branch support was assessed by the SH-like approximate likelihood ratio test[60].

For the subsequent phylodynamic and phylogeography analyses, we inspected the temporal signal by applying root-to-tip divergence regression against sampling time using TempEst v1.5.3[59] applied to the ML tree. The root-to-tip regression demonstrated that the datasets accumulated divergence evolved in a clock-like manner with correlation coefficients of 0.9651 for HRSV-A, and 0.9449 for HRSV-B.

Phylogeographic reconstructions

Bayesian phylodynamic analysis were performed, incorporating the previously inferred and inspected maximum likelihood (ML) tree as the starting topology, using BEAST package v1.10.5pre[60,19] with BEAGLE library v4.0.0[20] to enhance computational performance.

We used an Uncorrelated Lognormal Distribution (UCLD) relaxed-clock model with gamma-distributed among-site rate heterogeneity[23]. The Bayesian Skygrid coalescent model was utilized as a tree prior[22]. Markov Chain Monte Carlo (MCMC) runs performed in duplicate for 100 million iterations sampling every 50,000 steps. We used Tracer 1.7.2[24] to evaluate MCMC mixing, considering effective sample sizes (ESS), which were > 200 for each parameter. The runs were subsequently combined in LogCombiner[19], and a total of 1000 empirical trees were subsampled for each lineage to proceed to the next steps.

Based on the empirical trees, we selected monophyletic clades containing samples from Florida, with posterior support over 0.95, and with more than 20 samples. These are the clades identified in our set of analyses composed of sequences from the selected clades for HRSV-A (A.D.1 = 382, A.D.2 = 166, A.D.3 = 232, A.D.5 = 223, A.D = 330, and Florida = 26). We performed phylogeographic discrete trait analysis using an asymmetric substitution model and employing the Bayesian Stochastic Search Variable Selection (BSSVS)[24] to characterize the dissemination routes between global regions, United of States of America (USA) states, regions and Florida. The transition realizations were summarized between locations using a Markov Jumps approach[20,61,62]. The support of transmission routes was assessed using Bayes Factors for all pairwise comparisons. The Markov jumps were extracted using TreeMarkovJumpHistoryAnalyzer tool[26] and visualized using Circlize R package[27]. The Maximum Clade Credibility (MCC) tree was summarized with TreeAnnotator v1.10.5 pre-implemented in BEAST. The MCC tree visualization was generated using the ggtree package.

Estimation of *Ne* peak intervals before and after the COVID-19 bottleneck

To assess changes in temporal patterns of *Ne* population peaks before and after the onset of the COVID-19 pandemic (March 2020), we estimated the average time from *Ne* one peak to the next before and after the 2020 bottleneck using a custom *Python* (version 3.10) script (available from the authors upon request). Timestamps were converted from milliseconds to datetime format, and the data was split into pre- and post-pandemic periods. Ne peaks were identified automatically, rather than manually, from the median Bayesian Skygrid trajectory using the find peaks function from the SciPy library[63]. Local maxima were defined as time points at which the median *Ne* estimate exceeded neighbouring values in the Skygrid trajectory. The average time between consecutive Ne peaks was then calculated in both milliseconds and weeks for each period. This approach allowed us to quantify and compare peak periodicity across the two

time periods, providing insight into potential shifts in temporal dynamics associated with the pandemic. Because Skygrid smoothing can influence the detection of local maxima, these estimates were interpreted as relative changes in inferred bottleneck/expansion periodicity rather than exact timings of epidemiological events.

HRSV epitopes diversity

To evaluate entropy and diversity in G- and F-protein epitopes before versus after the COVID-19 pandemic, we used publicly available, experimentally tested, and curated epitopes described in the Immune Epitope Database (IEDB; https://www.iedb.org/)[28]. The IEDB search was performed on April 2, 2025, separately for B-cell and T-cell epitopes, using the following settings: "Respiratory syncytial virus (respiratory syncytial virus RS) (ID:12814, RSV)" and "Human respiratory syncytial virus (ID:11250, HRSV)" as the organism; Homo sapiens (human) as the host; "Infectious Disease" as disease data; and positive assays only. After data retrieval, epitopes were sorted according to HRSV F and G proteins. A total of three T-cell and 36 B-cell epitopes for the G protein, and 23 T-cell and 27 B-cell epitopes for the F protein, were included in the analyses. The pre- and post-pandemic HRSV-A A.D.1, A.D.2, A.D.3, and A.D.5 datasets, as well as the HRSV-B dataset, were evaluated separately. To calculate epitope entropy, Shannon entropy was first computed for each amino acid site using the formula[29]:

$$H = - \Sigma (\mathbf{p}_i * \log2(\mathbf{p}_i))$$

where $\boldsymbol{p_i}$ is the frequency of amino acid $\boldsymbol{i}$ at a given position, and the sum is calculated across all amino acids present at that position. Epitope entropy was then calculated as the sum of the entropy value across all amino acid position within the epitope. To assess whether entropy differed between pre- and post-pandemic datasets, the Wilcoxon signed-rank test was applied. For the F-protein entropy epitope distribution, Student's *t-test* was used when comparison of entropy distributions was appropriate. Because these analyses involved multiple exploratory comparisons across proteins, immune-response categories, viral lineages, and time periods, statistical results were interpreted cautiously. No formal multiple-comparison correction was applied, therefore, the entropy analyses were considered exploratory and hypothesis-generating. Figures and bar graphs were generated using RStudio version 2025.05.0+496[64].

To access the difference in genetic diversity of the complete genome coding regions per- versus post-pandemic, amino acid diversity was also quantified using Shannon entropy calculated per alignment position. Population-level diversity was summarized as the mean entropy across sites. Differences between pre- and post-pandemic mean entropy were evaluated using a non-parametric permutation test (10,000 permutations), with statistical significance assessed using a two-sided p-value. Uncertainty in the estimated difference was further quantified using non-parametric bootstrap resampling (10,000 replicates) to derive 95% confidence intervals.

Supplementary Figures and Tables

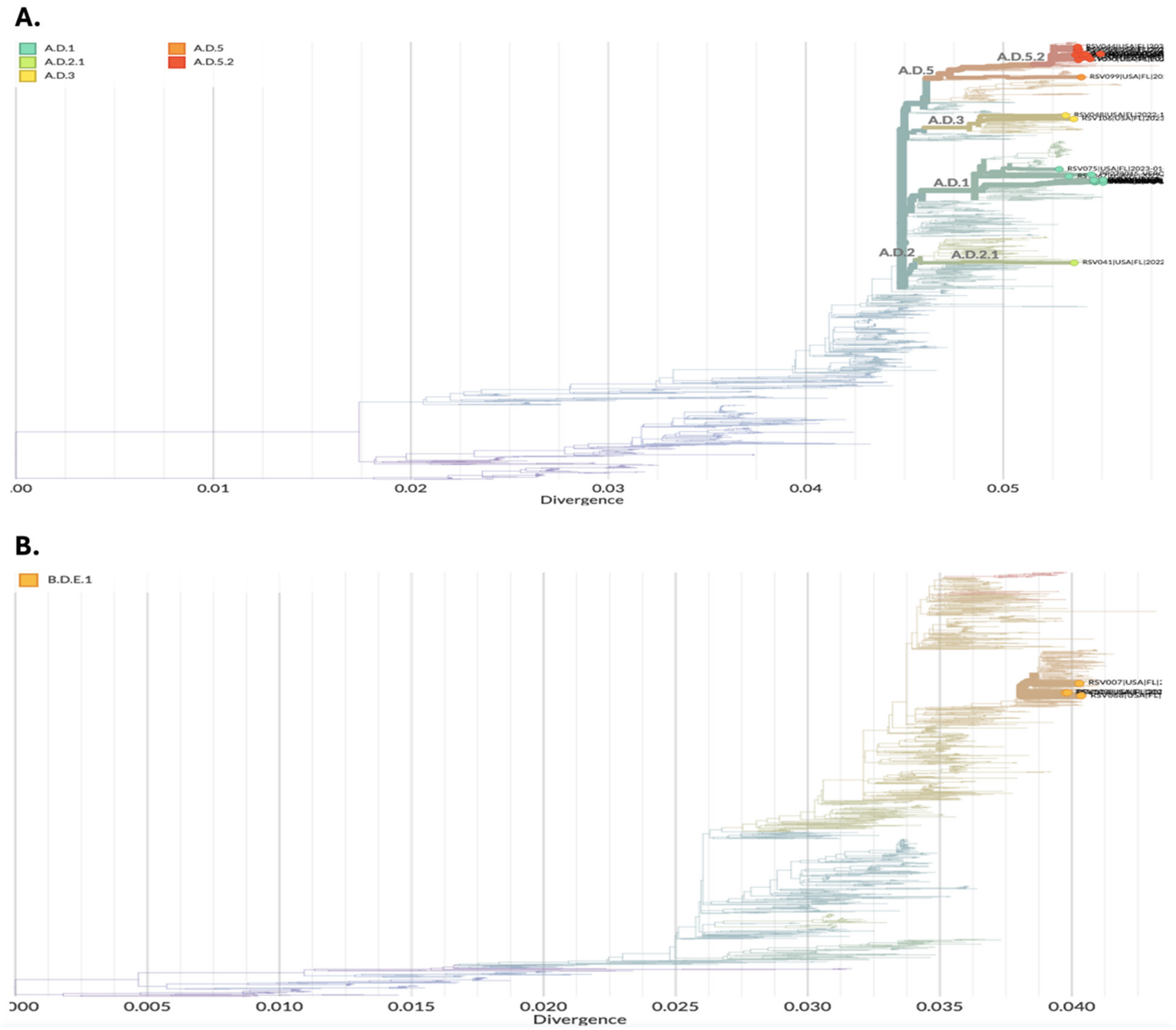

Figure S1. Nextclade HRSV clade classification for Florida sequences. Nextclade classification of Florida HRSV genomes showing(**A**) HRSV-A and (**B**) HRSV-B clade assignments. HRSV-A sequences were assigned to clades A.D.1 [green], A.D.2 [light green], A.D.3 [yellow], A.D.5 [orange], and A.D.5.2 [red], whereas all HRSV-B sequences were assigned to B.D.E.1 [light orange]. Colors denote Nextclade-defined clade membership.

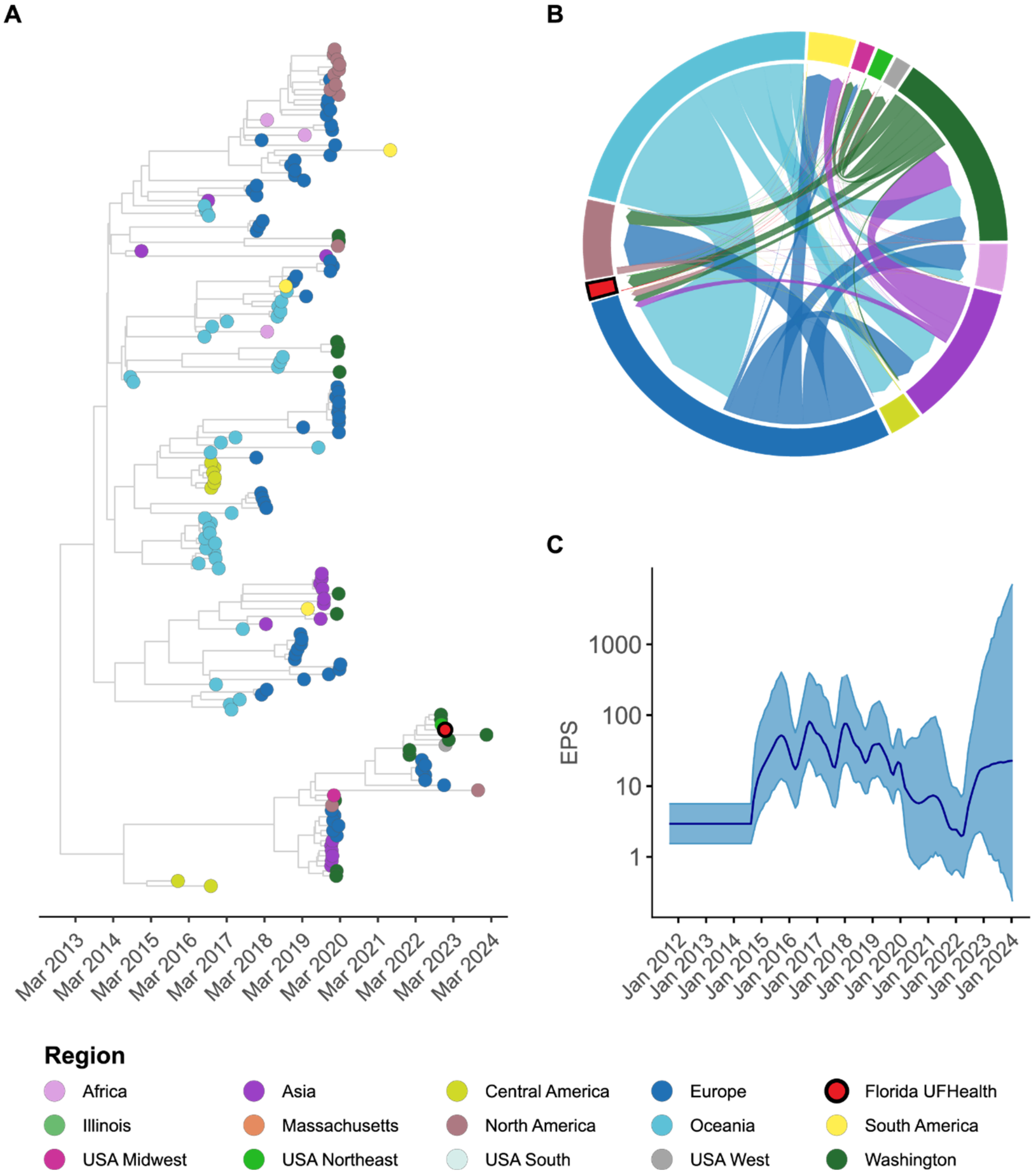

Figure S2. Phylogeographic reconstruction and Bayesian Skygrid analysis of HRSV-A Clade A.D.2. (**A**) Time-scaled MCC tree of HRSV-A Clade A.D.2 sequences, with branches and tips color-coded according to sampled or inferred geographic region as indicated in the figure color key. All Florida sequences originated from UFHealth (Alachua County, FL, USA) and are indicated by orange circles with black outlines. (**B**) Circular migration plot summarizing inferred viral movements among regions. Colors indicate geographic assignments, and ribbons represent inferred connections weighted by Markov jumps, which estimate transitions between geographic states. (**C**) Bayesian Skygrid plot showing temporal changes in relative effective population size, with the dark blue line indicating the median estimate and the light blue shaded region representing the uncertainty interval.

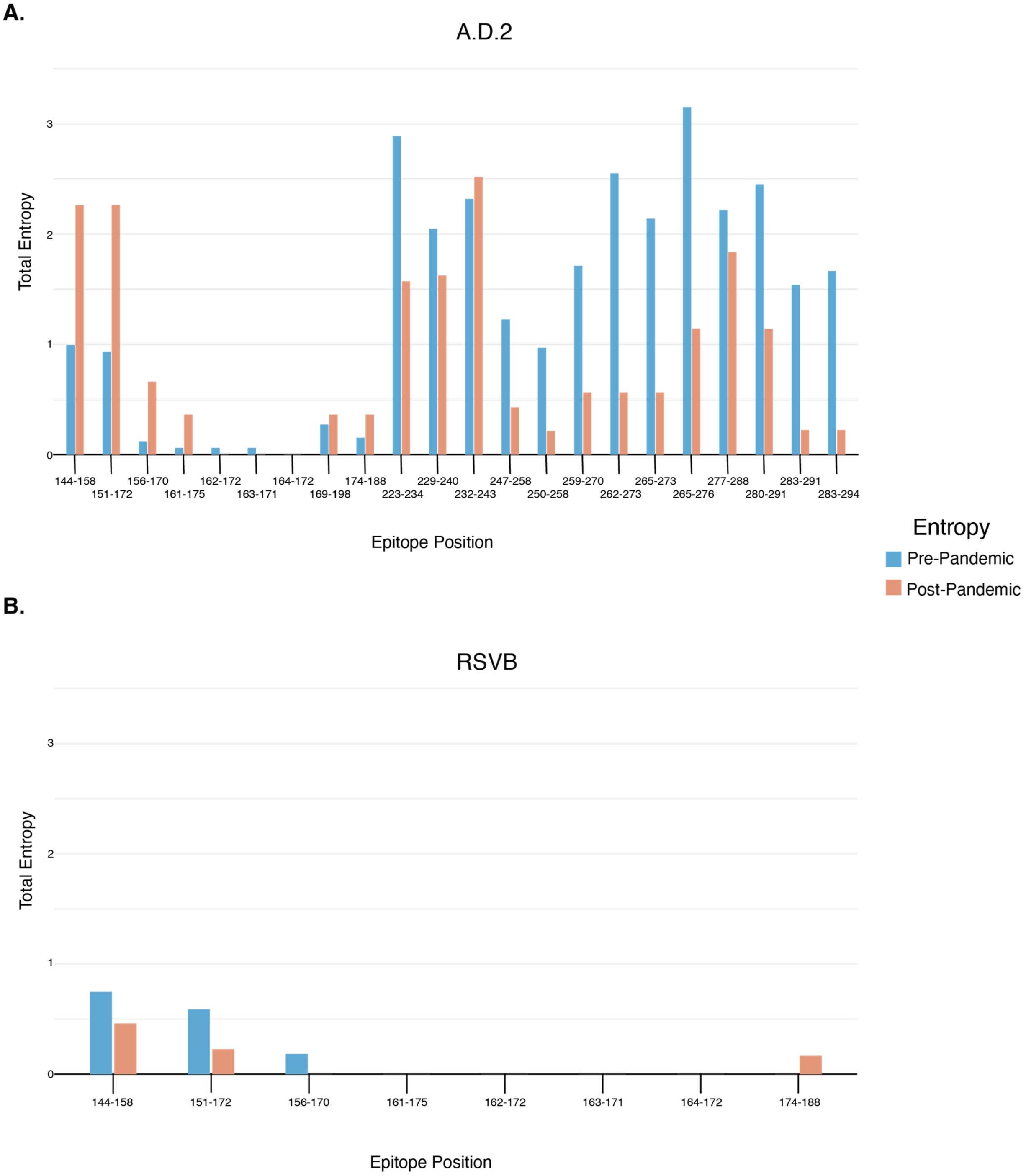

Figure S3. Entropy of HRSV-A G-protein epitopes before and after the COVID-19 pandemic. Total epitope entropy for HRSV-A A.D.2 (**A**) and HRSV-B (**B**) circulating in the United States before and after the onset of the COVID-19 pandemic. Blue bars represent pre-pandemic datasets, and red bars represent post-pandemic datasets. The x-axis indicates the start and end positions of each epitope according to the reference sequence NC_038235 (HRSV-A) and NC_001781 (HRSV-B), and the y-axis shows total entropy, with higher values indicating greater amino acid variability within the corresponding epitope region.

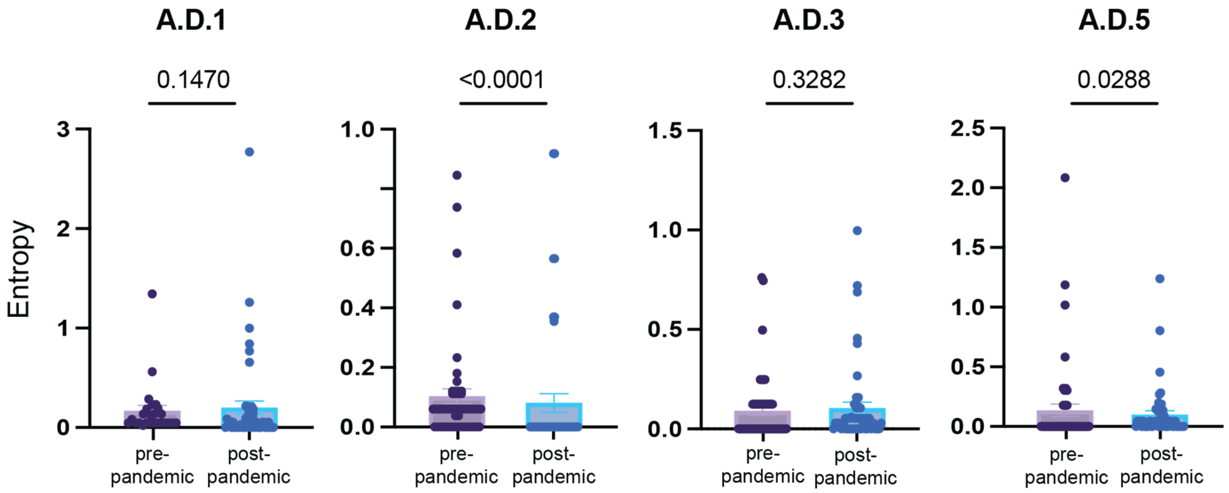

Figure S4. Dot plots with overlaid bars show entropy values (y-axis) calculated for four HRSV-A lineages (A.D.1, A.D.2, A.D.3, and A.D.5) from genomes before (pre-pandemic) and after (post-pandemic) the COVID-19 pandemic period. Each point represents an individual protein F epitope, with bars indicating central tendency and dispersion for each group. Statistical comparisons between pre-pandemic and post-pandemic groups for each lineage were performed using a non-parametric Mann–Whitney U test. Exact p-values are shown above each panel. Entropy is used as a measure of within-host viral genetic diversity, with higher values indicating increased sequence heterogeneity within the sampled viral population.

Table S1. HRSV sequences assembly metrics and genotype information

- Attached in excel format
